# High-Energy Enteral Nutrition in Infants After Complex Congenital Heart Surgery

**DOI:** 10.3389/fped.2022.869415

**Published:** 2022-07-13

**Authors:** Ping Ni, Xi Chen, Yueyue Zhang, Mingjie Zhang, Zhuoming Xu, Wenyi Luo

**Affiliations:** ^1^Department of Thoracic and Cardiovascular Surgery, Shanghai Children's Medical Center, School of Medicine, Shanghai Jiao Tong University, Shanghai, China; ^2^Nursing Department, Shanghai Children's Medical Center, School of Medicine, Shanghai Jiao Tong University, Shanghai, China

**Keywords:** congenital heart disease, pediatrics, malnutrition, enteral nutrition, randomized controlled trial

## Abstract

**Background:**

Malnutrition is common in complex congenital heart disease (CCHD). The purpose of this study was to compare the safety and efficacy of early initiation of high-energy enteral nutrition (EN) with regular energy EN in infants after surgery for CCHD.

**Methods:**

This is a subgroup analysis of a randomized controlled trial (RCT) which was conducted in the cardiac intensive care unit (CICU) of the largest pediatric heart center in China. Eighty children with CCHD after surgery were from two groups, the intervention group (*n* = 40) was given high-energy EN and the control group (*n* = 40) was given regular energy EN. We analyzed the effects of the two interventions on outcomes such as caloric attainment rate, gastrointestinal intolerance, duration of mechanical ventilation, and anthropometry at discharge.

**Results:**

There was no difference in the daily milk intake between the two groups, but the calorie intake (50.2 vs. 33.4, *P* < 0.001), protein intake (1.1 vs. 0.9, *P* < 0.001) and caloric attainment rate were higher in the intervention group (77.5 vs. 45.0%, *P* = 0.003). In addition, the incidence of pneumonia (*P* = 0.003) and duration of mechanical ventilation (*P* = 0.008) were less in the intervention group, and biceps circumference and triceps skinfold thickness at hospital discharge were greater than those in the control group (*P* < 0.001). We have not found statistical differences in gastrointestinal intolerance, glycemic fluctuations, incidence of pressure ulcers, length of CICU stay and postoperative hospital days between the two groups.

**Conclusions:**

Early initiation of high-energy EN may be safe and effective in infants after complex cardiac surgery. Low doses high-energy EN did not increase gastrointestinal intolerance or glycemic fluctuations and also improved post-operative nutrition by increasing caloric and protein intake without increasing fluid intake.

## Introduction

Congenital heart disease (CHD) is commonly defined as structural abnormalities of the heart and/or great vessels at birth. The increasing prevalence of CHD reported globally has made it the most common birth defect (1, 2). With advances in surgical and perioperative care techniques, survival rates for CHD have greatly improved, and malnutrition has become one of the main concerns of medical professionals. The incidence of acute and chronic malnutrition in children with CHD can be as high as 50% (3). Our previous survey of 3,253 children with CHD showed that the preoperative prevalence of underweight, stunting and wasting in children with CHD were 23.3, 23.3, and 14.3%, respectively, rising to approximately 25% in the postoperative period (4). It is evident that the situation of malnutrition in children with CHD is critical and requires urgent attention from medical professionals.

Numerous factors influence malnutrition in CHD, including genetic problems, improper feeding, cyanotic CHD, and pulmonary hypertension (4, 5). Surgical stress, mechanical ventilation, and vasoactive agents during hospitalization increase energy requirements (6), while fluid restriction, poor digestion, and malabsorption further exacerbate malnutrition (5, 7). All of these problems are exacerbated in critically ill children with CHD. Malnutrition not only increases the risk of postoperative infection, prolongs hospital stay, and increases mortality, but even affects the long-term growth and quality of life of patients and increases the socioeconomic burden (8–10).

Guidelines state that enteral nutrition (EN) is the best way to deliver nutrition (11). Early initiation of EN after surgery can reduce the duration of mechanical ventilation and ICU stay (12). High energy density EN can balance the conflict between fluid restriction and inadequate energy intake and reduce weight loss in CHD patients (13). However, we do not know how effective these regimens are in infants after open-heart surgery for complex CHD. Therefore, we conducted a subgroup analysis of an RCT with the aim of investigating the safety and efficacy of high-energy EN in infants with CCHD. Our hypothesis was that high-energy EN would not increase gastrointestinal intolerance compared to regular energy EN and would improve outcomes such as caloric attainment rate, nutritional outcomes and ICU outcomes without increasing fluid intake.

## Materials and Methods

### Study Design

This study is a subgroup analysis of a randomized controlled trial with a sample size of 244 cases. The study site was the CICU of Shanghai Children's Medical Center, the largest pediatric heart center in China, and the study period was from July 2016 to July 2018. This study was approved by the Ethics Committee of Shanghai Children's Medical Center (SCMCIRB-K2015040) and was registered on ClinicalTrials.gov PRS (NCT 04609358). All guardians of the patients were informed and signed the informed consent form.

### Participants

The sample size for this subgroup analysis was 80 cases. All children in the RCT who met the following criteria were included: (i) age <6 months, (ii) RACHS-1 score ≥3, (iii) post-open heart surgery for CHD, (iv) moderate or severe malnutrition as determined by *z*-scores established by the World Health Organization (WHO), including weight-for-height *z*-score (WHZ), weight-for-age *z*-score (WAZ) or height-for-age *z*-score (HAZ) <-2 (14). Exclusion criteria were as follows: (i) children receiving total parenteral nutrition postoperatively, (ii) combination of genetic problems such as D-george syndrome, or diseases causing nutritional disorders such as gastrointestinal malformations, (iii) milk protein allergy, and (iv) unwillingness of the patient's guardian to participate in this study.

### Procedures

Before the start of the study, all physicians and nurses in the CICU were trained to understand the purpose of this study, the implementation process, and the management of special cases. The patient's condition was assessed by physicians within 6 h after surgery, and the chief physician issued a medical prescription for EN (including the type of milk, the amount of milk, the frequency of feeding and the feeding method) as soon as the condition allowed. The intervention group was given high energy milk powder (100 kcal/100 ml, protein: 2–3 g/100 ml, osmotic pressure: 281–340 mOsm/L) and the control group was given regular energy milk powder (67–82 kcal/100 ml, protein: 1.8–2.3 g/100 ml, osmotic pressure: 185–340 mOsm/L). A feeding advancement schedule was used: initiate feeding at 1–2 ml/kg/h for 6–24 hours after surgery and increase by 1–2 ml/kg/h per day, if tolerated, until target energy supply prior to discharge is achieved.

All mechanically ventilated infants were fed through a nasogastric tube, and after extubation, if the infants could eat through the mouth, they were changed to oral feeding. If the infant vomited >3 times in 24 h or showed symptoms of feeding intolerance such as positive fecal occult blood, the feeding was suspended and the doctor in charge chose to continue feeding or change to parenteral nutrition according to the review results.

The hospital nutrition department prepared the milk powder according to the medical prescription and transported it to CICU by special personnel, and then the nurses gave the infants EN according to the prescription. All infants were provided with formula by the hospital during the study period and did not receive mother's milk. The whole intervention process continued until the patient was discharged from the hospital.

### End Points

The primary outcome of this study was the caloric attainment rate of enteral feeding during CICU. The resting energy expenditure (REE) of infants was estimated according to the Schofeld formula (15, 16) recommended by the European Society of Pediatric and Neonatal Intensive Care (ESPNIC), and caloric intake of enteral feeding of no <80% of the REE was defined as caloric attainment rate is met.

Secondary outcomes were calorie and protein intake, duration of mechanical ventilation, duration of ICU stay, duration of postoperative hospital stay, pneumonia, pressure ulcers, mean blood glucose and its fluctuations within 5 days postoperatively, biceps circumference and triceps skinfold thickness at discharge, and feeding-related complications (including diarrhea, fecal occult blood). Pneumonia was defined by the presence of a new or evolving infiltrate on chest X-ray combined with corresponding clinical manifestations (17).

Arterial blood gases were measured every 4 h during the infant's postoperative stay in the CICU, so there were six blood glucose data per day. Blood glucose fluctuations were expressed as the largest amplitude of glycemic excursions (LAGE), which is the difference between the highest and lowest blood glucose during the day (18).

### Data Collection

The infants' general demographic data, intraoperative data, postoperative diarrhea, fecal occult blood, duration of mechanical ventilation, blood glucose, and ICU length of stay were obtained from the hospital information system. Height, weight, biceps circumference, and triceps skinfold thickness were measured by trained researchers.

The *z*-score of infants was calculated using WHO's Anthro v3.2.2 software (19). A *z*-score <-2 was defined as malnutrition, including HAZ <-2 (stunting), WAZ <-2 (underweight) or WHZ <-2 (wasting).

### Statistical Analysis

All data analyses were performed using the SPSS 23.0 (IBM, Armonk, NY, USA). Shapiro-Wilk normality test was performed on all data. Continuous data were expressed as mean and standard deviation or median and its quartiles, and *t*-test, Mann–Whitney *U*-test, repeated measures ANOVA, and analysis of covariance were used for comparison between groups. Categorical data were expressed as frequencies and composition ratios, and comparisons between groups were made by Chi-square test or Fisher's exact probability. *P* < 0.05 was considered statistically significant.

## Results

A total of 255 infants were recruited, 244 of whom completed the randomized controlled trial. Of these 244 infants, 80 infants had a RACHS-1 score ≥3 and were therefore included in this study, 40 in the intervention and 40 in the control group. All the patients completed the study and were successfully discharged with no deaths. 60% of the patients in the intervention group were male, and 75% of the patients in the control group were male. The median RACHS-1 score in both groups was 3. The baseline characteristics of the patients in both groups are shown in Table 1, and the major surgical procedures and diagnosis are shown in Supplementary Material 1.

**Table 1:** Baseline characteristics of the participants.

	**Intervention group**	**Control group**	***P*-Value**
	**(*n* = 40)**	**(*n* = 40)**	
Gender, male	24 (60%)	30 (75%)	0.152
Age (days)	88.5 ± 51.8	87.5 ± 46.4	0.924
Biceps circumference (cm)	10.6 ± 1.6	10.4 ± 1.6	0.665
Triceps skinfold thickness (mm)	7.3 ± 2.4	6.7 ± 2.4	0.302
WHZ	−1.5 ± 1.8	−1.3 ± 2.0	0.654
WAZ	−2.8 ± 1.1	−2.7 ± 1.1	0.705
HAZ	−2.0 ± 2.0	−2.1 ± 1.6	0.712
Preoperative intubation	7 (17.5%)	8 (20.0%)	0.775
Delayed sternal closure	8 (20.0%)	9 (22.5%)	0.785
Cardiopulmonary bypass time (min)	86.2 ± 46.2	103.4 ± 62.4	0.165
Aortic clamping time (min)	55.0 ± 32.2	64.8 ± 38.6	0.221
RACHS-1	3 (3, 4)	3 (3, 4)	0.869

*Data are n (%), mean ± SD or median (P_25_, P_75_), unless otherwise specified*.

*WHZ, weight for height z-score; WAZ, weight for age z-score; HAZ, height for age z-score; RACHS-1, risk adjustment for congenital heart surgery 1 score; SD, standard deviation*.

### Primary Outcome

There was no statistically significant difference in the daily milk intake in the intervention group compared to the control group (50.2 vs. 49.8 ml/kg/day, *P* = 0.881). With this in mind, the intervention group had a statistically significant higher caloric attainment rate of enteral feeding than the control group (77.5 vs. 45.0%, *P* = 0.003) (Table 2).

**Table 2:** Feeding related outcomes and clinical outcomes.

	**Intervention group**	**Control group**	***P*-Value**
	**(*n* = 40)**	**(*n* = 40)**	
**Primary outcome**			
Caloric attainment rate	31 (77.5%)	18 (45.0%)	0.003
**Secondary outcomes**			
Milk intake (ml/kg/day)	50.2 ± 12.5	49.8 ± 11.3	0.881
Average calorie intake (kcal/kg/day)	50.2 ± 12.5	33.4 ± 7.6	<0.001
Average protein intake (g/kg/day)	1.1 ± 0.4	0.9 ± 0.2	0.011
Diarrhea	4 (10.0%)	2 (5%)	0.675
Fecal occult blood	1 (2.5%)	5 (12.5%)	0.201
Mechanical ventilation (h)	35.0 (14.9, 88.0)	81.0 (34.7, 136.3)	0.008
ICU stay (day)	7.0 (4.3, 8.8)	7.0 (5.3, 11.5)	0.263
Hospital stay after operation (day)	13.5 (10.3, 19.8)	15.0 (10.0,19.0)	0.579
Pneumonia	1 (2.5%)	10 (25.0%)	0.003
Pressure ulcer	2 (5.0%)	5 (12.5%)	0.432
Chylothorax	5 (12.5%)	5 (12.5%)	1.000

*Data are n (%), mean ± SD or median (P_25_, P_75_), unless otherwise specified*.

*ICU, intensive care unit; SD, standard deviation*.

### Secondary Outcomes

The average caloric intake (50.2 vs. 33.4 kcal/kg/day, *P* < 0.001) and protein intake (1.1 vs. 0.9 g/kg/day, *P* = 0.011) of the intervention group were higher than those of the control group. There were no differences between the two groups in terms of diarrhea or fecal occult blood. Patients in the intervention group had a shorter duration of mechanical ventilation (35 vs. 81 h, *P* = 0.008) and a lower incidence of pneumonia (2.5 vs. 25%, *P* = 0.003) than the control group as shown in Table 2. Adjusted means showed that biceps circumference (10.7 vs. 9.7 cm), triceps skinfold thickness (7.2 vs. 5.9 mm) were greater in the trial group than in the control group at discharge, with statistically significant differences (all *P*-values were <0.001; Table 3)

**Table 3:** Anthropometry at discharge.

	**Intervention group**	**Control group**	***P*-Value**
	**(*n* = 40)**	**(*n* = 40)**	
Biceps circumference (cm)	10.7 (10.5, 11.0)	9.7 (9.4, 9.9)	<0.001
Triceps skinfold thickness (mm)	7.2 (6.9, 7.4)	5.9 (5.6, 6.1)	<0.001

*Data were adjusted according to preoperative data, and were expressed by means (95% CI)*.

**Figure 1:**
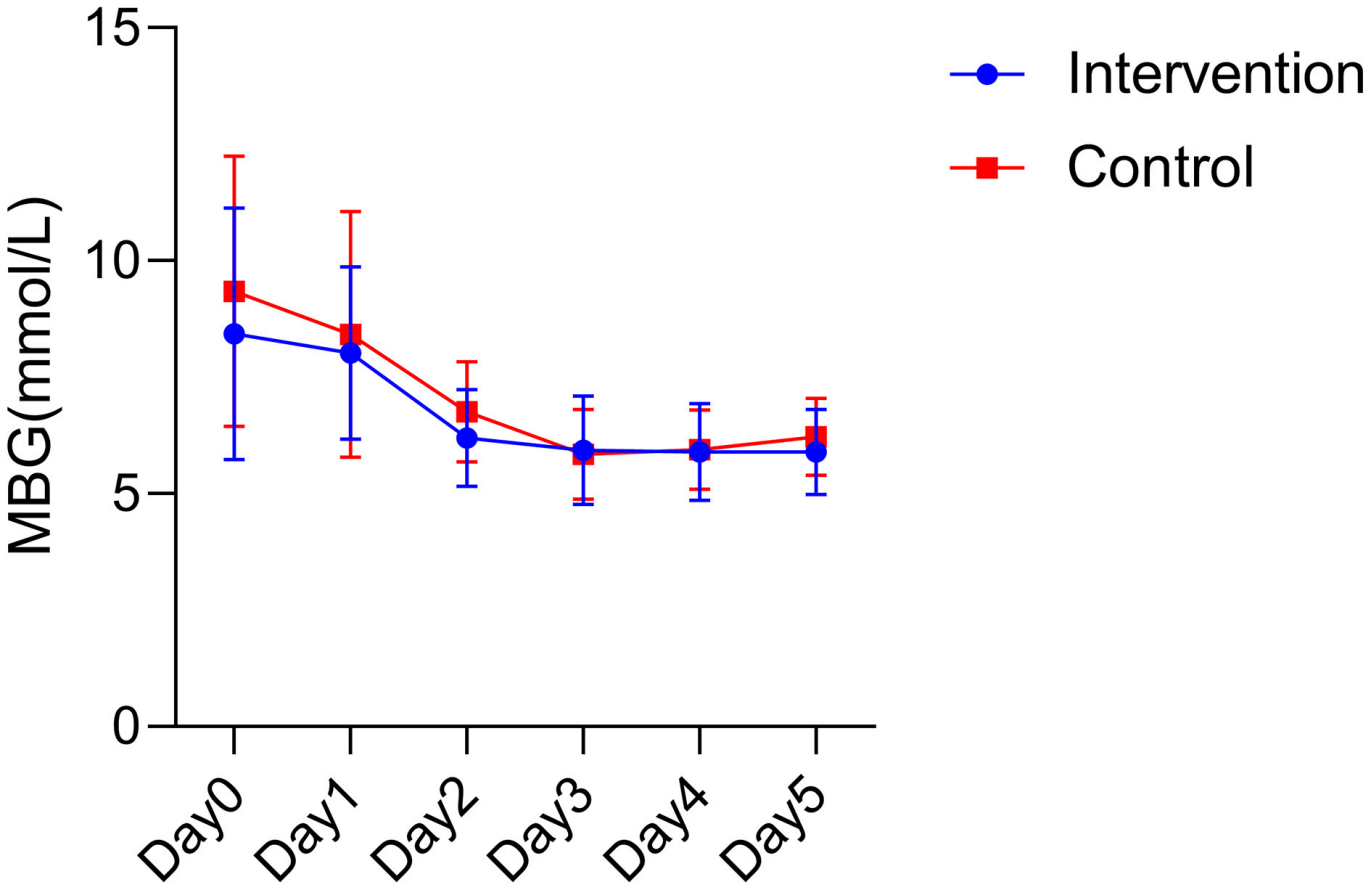
Postoperative mean blood glucose between the two groups. MBG, mean blood glucose.

Analysis of variance showed that there was no statistical difference in the mean blood glucose between the two groups (*F* = 2.099, *P* = 0.153). Time had an effect on mean blood glucose in the postoperative period, with the mean blood glucose in the intervention group showing a tendency to first decrease and then gradually stabilize within 5 days after surgery, and the mean blood glucose in the control group showing a tendency to first decrease and then mildly increase within 5 days after surgery. An interaction effect between intervention and time was not found (*F* = 0.558, *P* = 0.604; Figure 1).

**Figure 2:**
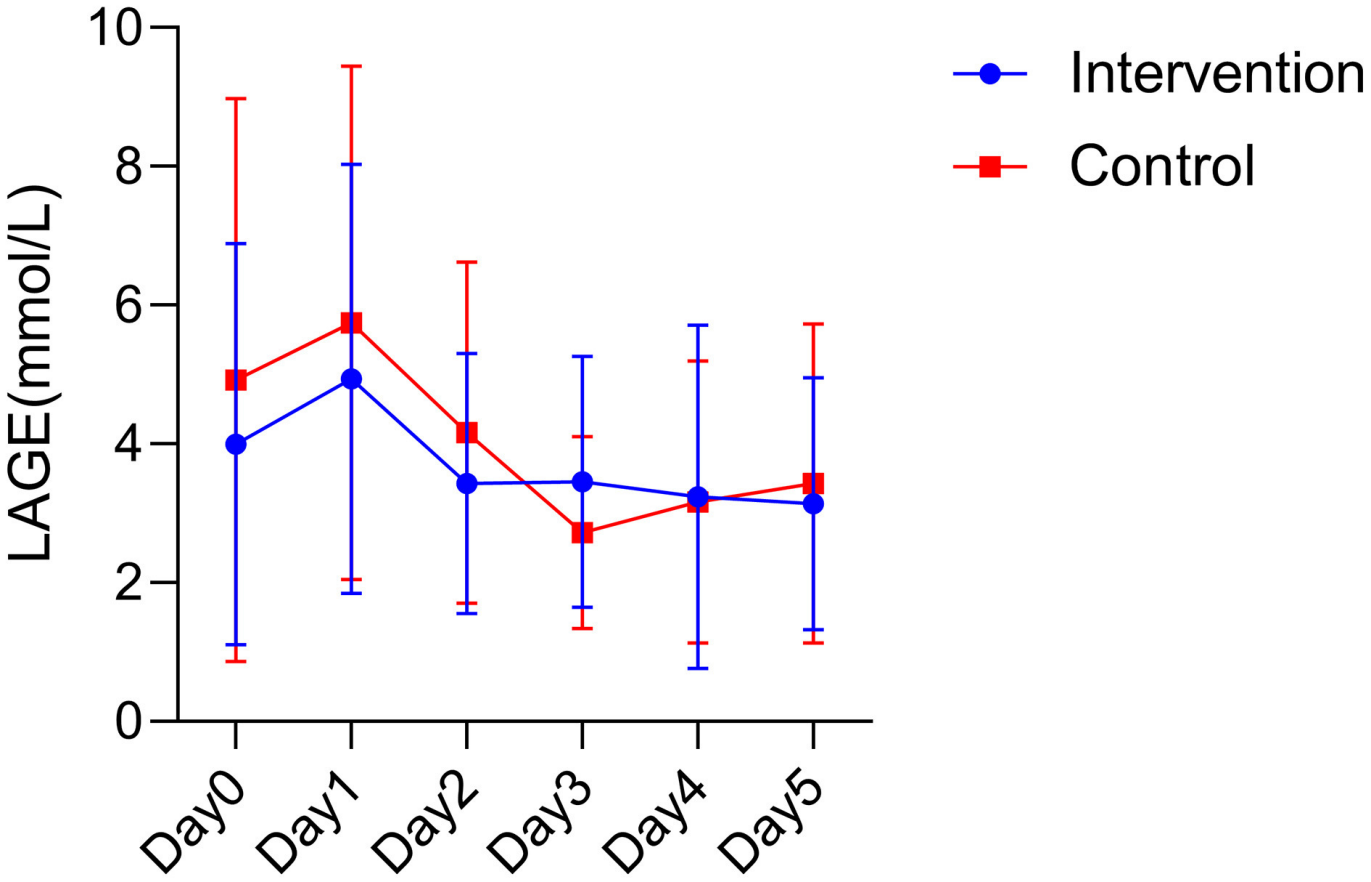
Postoperative blood glucose fluctuations between the two groups. LAGE, largest amplitude of glycemic excursions.

The blood glucose fluctuations of the two groups at different times after surgery were basically in a gradual and decreasing trend in the intervention group, while the blood glucose fluctuations in the control group were more variable. However, no statistical difference was found between the two groups in postoperative blood glucose fluctuations (*F* = 0.984, *P* = 0.326; Figure 2).

## Discussion

In this subgroup analysis of an RCT, we compared the safety and efficacy of high-energy EN with regular energy EN in children with CCHD. The results show that early initiation of high-energy nutrition does not increase gastrointestinal intolerance, while also improving caloric attainment rate without increasing fluid intake and improving nutritional outcomes and ICU outcomes in CCHD infants.

Our study did not find a statistical difference in the incidence of diarrhea and fecal occult blood between the two groups, suggesting that critical postoperative infants with CHD can tolerate high-energy nutrition. Two other studies with similar results to the present study have also confirmed that high-energy formula does not increase gastrointestinal adverse effects such as vomiting and diarrhea in patients, nor cardiac, renal and hepatic adverse effects (20, 21). Even if the patients in the study by Zhang et al. (13) experienced a small amount of abdominal distention and gastric retention, they could be relieved by gastroprokinetic drugs without affecting the feeding process.

According to the guidelines, energy intake should not exceed resting energy expenditure in critically ill children in the acute phase. Therefore, in this study we defined caloric attainment as the intake of energy up to 80% of REE. The results of the study showed that patients in the intervention group had a higher rate of caloric attainment without increasing fluid intake. In the management of CHD, fluid intake usually needs to be controlled to reduce multi-organ dysfunction caused by the fluid overload (22, 23). However, inadequate intake is an important influencing factor of postoperative malnutrition, leading to a high prevalence of postoperative malnutrition in CHD. Faced with such a paradox, high-energy milk powder has a significant advantage. The high-energy milk powder used in the experimental group had an energy density of 100 kcal/100 ml, which was on average 25.5 kcal/100 ml higher than that of the control group, and it could provide more calories and protein for patients with the same amount of fluid intake, which facilitated the improvement of malnutrition in patients.

Nutritional assessment includes dietary history, anthropometry, functional status, and physical examination with a nutritional focus (11). Anthropometric measurements have the advantage of being easy to perform and non-invasive. In this study, we measured biceps circumference and triceps skinfold thickness at patient discharge to reflect short-term nutritional status. The results showed that patients in the high-energy nutrition group had greater biceps circumference and triceps skinfold thickness at discharge than the control group, suggesting that high-energy nutrition is more beneficial than regular nutrition in improving malnutrition. There is a significant correlation between nutritional status and the prognosis of critically ill children (24, 25), which was mainly reflected in this study by shorter duration of mechanical ventilation and lower incidence of pneumonia in the intervention group. This is because earlier extubation prevents pneumonia (26); it is also possible that children with poor nutritional status have a poor inflammatory response and are therefore more susceptible to pneumonia, as other studies have confirmed (27, 28). However, the findings on whether high-energy nutrition shortens the length of hospital stay have not been uniform. Some studies found that high-energy nutrition shortens the length of hospital stay (12, 29), while some studies, in agreement with the results of the present study, did not find any difference in the length of ICU stay or postoperative hospital stay between the intervention and control groups (13). Since all infants in this study had complex CHD and were young, for safety reasons, the intervention group needed to adjust and observe the function of other organs and systems after extubation, such as the heart and kidneys, until they were stabilized and then transferred out of the ICU. Therefore, there was no significant difference between the two groups in terms of ICU stay.

Blood glucose fluctuations have a significant correlation with the prognosis of critically ill patients, and mortality is higher in patients with high blood glucose fluctuations (30). Although high-energy nutrition contains more protein, fat and carbohydrates, there was no difference in blood glucose fluctuations between the two groups of patients in this study. We also observed essentially similar trends in glycemic changes in both groups, with the highest on the postoperative day and a gradual decline thereafter. This trend may be related to hyperglycemia caused by stress response (31). Patients had the highest stress response on the postoperative day and therefore had the highest blood glucose. This phenomenon indicates that high energy nutrition did not cause fluctuations in the condition of the patients, which may reflect the safety of high energy nutrition. Interestingly, we also found that the blood glucose in the intervention group was always closer to the normal range than in the control group, and there was a slight increase in blood glucose in the control group starting on postoperative day 4 and gradually rising above the normal value. Further studies could be done in the future to investigate the underlying mechanism of this phenomenon.

A limitation of this study is that the population studied are generally not the most at-risk children with CHD and malnutrition, which are neonates or younger infants, making the results may not be applicable to all patients. Then, it is worth noting that this study did not stratify patients according to underlying disease, surgical events, or nutritional status, which could lead to selection bias. In addition, we did not compare the nitrogen balance of the two groups. Finally, due to the limitations of the conditions, indirect calorimetry was not used to measure the REE of each patient, but the formula recommended by the guidelines was used to estimate the REE, which may have some influence on the accuracy of energy estimation.

In conclusion, our study shows that early administration of high-energy EN was safe and effective in complex CHD infants (RACHS-1 ≥3). Low doses high-energy EN did not increase gastrointestinal complications and also improved calorie intake, protein intake and the caloric attainment rate. In addition, high-energy EN improved the nutritional status of patients at hospital discharge and may be associated with shorter duration of mechanical ventilation and a lower incidence of pneumonia. In terms of blood glucose, the effects of high-energy EN interventions and regular EN interventions were similar, and this similar effect was mainly reflected in mean blood glucose and blood glucose fluctuations.

## Data Availability Statement

The original contributions presented in the study are included in the article/supplementary material, further inquiries can be directed to the corresponding authors.

## Ethics Statement

The studies involving human participants were reviewed and approved by the Ethics Committee of Shanghai Children's Medical Center. Written informed consent to participate in this study was provided by the participants' legal guardian/next of kin. Written informed consent was obtained from the individual(s), and minor(s)' legal guardian/next of kin, for the publication of any potentially identifiable images or data included in this article.

## Author Contributions

ZX, WL, and MZ: conceptualized, designed the study, and revised the manuscript. PN and XC: data collection and drafted the initial manuscript. YZ: coordinated and supervised data collection. All authors approved the final manuscript as submitted and agree to be accountable for all aspects of the work.

## Conflict of Interest

The authors declare that the research was conducted in the absence of any commercial or financial relationships that could be construed as a potential conflict of interest.

## Publisher's Note

All claims expressed in this article are solely those of the authors and do not necessarily represent those of their affiliated organizations, or those of the publisher, the editors and the reviewers. Any product that may be evaluated in this article, or claim that may be made by its manufacturer, is not guaranteed or endorsed by the publisher.

## References

[1] Pierpont ME Brueckner M Chung WK Garg V Lacro RV McGuire AL. Genetic basis for congenital heart disease: revisited: a scientific statement from the American Heart Association. Circulation. (2018) 138:e653–711. 10.1161/CIR.0000000000000606 30571578 PMC6555769

[2] Liu Y Chen S Zühlke L Blank G Choy M Li N. Global birth prevalence of congenital heart defects 1970-2017: updated systematic review and meta-analysis of 260 studies. Int J Epidemiol. (2019) 48:455–63. 10.1093/ije/dyz009 30783674 PMC6469300

[3] Toole BJ Toole LE Kyle UG Cabrera AG Orellana RA Coss-Bu JA. Perioperative nutritional support and malnutrition in infants and children with congenital heart disease. Congenit Heart Dis. (2014) 9:15–25. 10.1111/chd.12064 23602045

[4] Zhang M Wang L Huang R Sun C Bao N Xu Z. Risk factors of malnutrition in Chinese children with congenital heart defect. BMC Pediatr. (2020) 20:213. 10.1186/s12887-020-02124-7 32404077 PMC7218652

[5] Argent AC Balachandran R Vaidyanathan B Khan A Kumar RK. Management of undernutrition and failure to thrive in children with congenital heart disease in low- and middle-income countries. Cardiol Young. (2017) 27(S6):S22–30. 10.1017/S104795111700258X 29198259

[6] Trabulsi JC Irving SY Papas MA. Hollowell C Ravishankar C Marino BS. Total energy expenditure of infants with congenital heart disease who have undergone surgical intervention. Pediatr Cardiol. (2015) 36:1670–9. 10.1007/s00246-015-1216-3 26092599

[7] Leong AY Cartwright KR Guerra GG Joffe AR Mazurak VC Larsen BMK. Canadian survey of perceived barriers to initiation and continuation of enteral feeding in PICUs. Pediatr Crit Care Med. (2014) 15:e49–55. 10.1097/PCC.0000000000000016 24196008

[8] Ross FJ Radman M. Jacobs ML Sassano-Miguel C Joffe DC Hill KD. Associations between anthropometric indices and outcomes of congenital heart operations in infants and young children: an analysis of data from the Society of Thoracic Surgeons Database. Am Heart J. (2020) 224:85–97. 10.1016/j.ahj.2020.03.012 32353587

[9] Lim CYS Lim JKB Moorakonda RB Ong C Mok YH Allen JC. The impact of pre-operative nutritional status on outcomes following congenital heart surgery. Front Pediatr. (2019) 7:429. 10.3389/fped.2019.00429 31709202 PMC6820300

[10] Batte A Lwabi P Lubega S Kiguli S Otwombe K Chimoyi L. Wasting, underweight and stunting among children with congenital heart disease presenting at Mulago hospital, Uganda. BMC Pediatr. (2017) 17:10. 10.1186/s12887-017-0779-y 28077108 PMC5225644

[11] Mehta NM Skillman HE Irving SY Coss-Bu JA Vermilyea S Farrington EA. Guidelines for the provision and assessment of nutrition support therapy in the pediatric critically ill patient: society of critical care medicine and American Society for Parenteral and Enteral Nutrition. JPEN J Parenter Enteral Nutr. (2017) 41:706–42. 10.1177/0148607117711387 28686844

[12] Kalra R. Vohra R Negi M Joshi R Aggarwal N Aggarwal M. Feasibility of initiating early enteral nutrition after congenital heart surgery in neonates and infants. Clin Nutr ESPEN. (2018) 25:100–2. 10.1016/j.clnesp.2018.03.127 29779802

[13] Zhang H Gu Y Mi Y Jin Y Fu W Latour J. High-energy nutrition in paediatric cardiac critical care patients: a randomized controlled trial. Nurs Crit Care. (2019) 24:97–102. 10.1111/nicc.12400 30548121

[14] World Health Organization. WHO Child Growth Standards: Length/Height-for-Age, Weight-for-Age, Weight-for-Length, Weight-for-Height and Body Mass Index-for-Age: Methods and Development. Geneva: World Health Organization (2006).

[15] Tume LN Valla FV Joosten K Chaparro CJ Latten L Marino LV. Nutritional support for children during critical illness: European Society of Pediatric and Neonatal Intensive Care (ESPNIC) metabolism, endocrine and nutrition section position statement and clinical recommendations. Intensive Care Med. (2020) 46:411–25. 10.1007/s00134-019-05922-5 32077997 PMC7067708

[16] Schofield WN. Predicting basal metabolic, new standards and review of previous work. Hum Nutr Clin Nutr. (1985) 39(C Suppl 1):5–41. 4044297

[17] Poelaert J Haentjens P Blot S. Association among duration of mechanical ventilation, cuff material of endotracheal tube, and postoperative nosocomial pneumonia in cardiac surgical patients: a prospective study. J Thorac Cardiovasc Surg. (2014) 148:1622–7. 10.1016/j.jtcvs.2014.05.085 25127550

[18] Shen L Ao L Xu H Shi JF You D Yu XW. Poor short-term glycemic control in patients with type 2 diabetes impairs the intestinal mucosal barrier: a prospective, single-center, observational study. BMC Endocr Disord. (2019) 19:29. 10.1186/s12902-019-0354-7 30849982 PMC6408809

[19] Mitting R Marino L Macrae D Shastri N Meyer R Pathan N. Nutritional status and clinical outcome in postterm neonates undergoing surgery for congenital heart disease. Pediatr Crit Care Med. (2015) 16:448–52. 10.1097/PCC.0000000000000402 25828781

[20] Akiko TF Mio M Teruyoshi A. Effect of a high density formula on growth and safety in congenital heart disease infants. E Spen Eur E J Clin Nutr Metab. (2010) 5:e281–3. 10.1016/j.eclnm.2010.10.002

[21] Scheeffer VA Ricachinevsky CP. Freitas AT Salamon F Rodrigues FFN Brondani TG. Tolerability and effects of the use of energy-enriched infant formula after congenital heart surgery: a randomized controlled trial. JPEN J Parenter Enteral Nutr. (2020) 44:348–54. 10.1002/jpen.1530 30900268

[22] Hanot J Dingankar AR Sivarajan VB Sheppard C Cave D Guerra GG. Fluid management practices after surgery for congenital heart disease: a worldwide survey. Pediatr Crit Care Med. (2019) 20:357–64. 10.1097/PCC.0000000000001818 30950987

[23] Hazle MA Gajarski RJ Yu S Donohue J Blatt NB. Fluid overload in infants following congenital heart surgery. Pediatr Crit Care Med. (2013) 14:44–9. 10.1097/PCC.0b013e3182712799 23249789 PMC3668443

[24] Marwali EM Darmaputri S Somasetia DH Sastroasmoro S Haas N Portman MA. Does malnutrition influence outcome in children undergoing congenital heart surgery in a developing country? Paediatr Indones. (2015) 55:109–16. 10.14238/pi55.2.2015.109-16

[25] Compher C Chittams J Sammarco T Nicolo M Heyland DK. Greater protein and energy intake may be associated with improved mortality in higher risk critically ill patients: a multicenter, multinational observational study. Crit Care Med. (2017) 45:156–63. 10.1097/CCM.0000000000002083 28098623

[26] Strobel RJ Harrington SD Hill C Thompson MP Cabrera L Theurer P. Evaluating the impact of pneumonia prevention recommendations after cardiac surgery. Ann Thorac Surg. (2020) 110:903–10. 10.1016/j.athoracsur.2019.12.053 32035918 PMC7646315

[27] Morgan G. What if any, is the effect of malnutrition on immunological competence? Lancet. (1997) 349:1693–5. 10.1016/S0140-6736(96)12038-9 9186397

[28] United Nations Children's Fund (UNICEF). Childhood Pneumonia: Everything you Need to Know | UNICEF. (2020). Available online at: https://www.unicef.org/stories/childhood-pneumonia-explained (accessed January 29, 2022).

[29] Pillo-Blocka F Adatia I Sharieff W. MccrindleBW Zlotkin S. Rapid advancement to more concentrated formula in infants after surgery for congenital heart disease reduces duration of hospital stay: a randomized clinical trial. J Pediatr. (2004) 145:761–6. 10.1016/j.jpeds.2004.07.043 15580197

[30] Hsu CW Sun SF Lin HS. Glycemic variability in critically ill patients: risk factors and association with mortality. Chin Med J. (2020) 133:1255–6. 10.1097/CM9.0000000000000686 32433063 PMC7249721

[31] Galindo RJ Fayfman M Umpierrez GE. Perioperative management of hyperglycemia and diabetes in cardiac surgery patients. Endocrinol Metab Clin North Am. (2018) 47:203–22. 10.1016/j.ecl.2017.10.005 29407052 PMC5805476

