# Supplementary material for: High-Energy Enteral Nutrition in Infants After Complex Congenital Heart Surgery
Source: Front Pediatr. 2022 Jul 13;10:869415. doi: 10.3389/fped.2022.869415 (PMC9326115; doi:10.3389/fped.2022.869415)
Supplement: Supplementary file 1 [file Table_1.pdf]

**Supplementary Material 1.** Major surgical procedures and diagnosis of the participants

| ***Surgical procedures*** | ***n(%)*** | ***Diagnosis*** | ***n(%)*** |
|---|---|---|---|
| COA repair + VSD membranous repair | 17 (21.3) | COA | 17 (21.3) |
| Valvuloplasty | 10 (12.5) | Valvular disease | 11 (13.8) |
| DORV repair | 9 (11.3) | TGA | 11 (13.8) |
| AVSD repair | 8 (10.0) | DORV | 10 (12.5) |
| ASO + VSD membranous repair | 7 (8.8) | AVSD | 8 (10.0) |
| TAPVC repair | 6 (7.5) | TAPVC | 6 (7.5) |
| HAA repair + VSD membranous repair | 5 (6.3) | HAA | 5 (6.3) |
| ASO | 4 (5.0) | IAA | 3 (3.8) |
| Blalock Taussig | 3 (3.8) | PA | 2 (2.5) |
| Pulmonary artery banding | 3 (3.8) | Tetralogy of Fallot | 2 (2.5) |
| IAA repair | 3 (3.8) | ALCAPA | 1 (1.3) |
| Tetralogy repair + PA repair | 1 (1.3) | Truncus arteriosus | 1 (1.3) |
| Truncus repair | 1 (1.3) | Hemitruncus | 1 (1.3) |
| Atrial septectomy | 1 (1.3) | VSD | 1 (1.3) |
| Conduit RV-pulmonary artery | 1 (1.3) | Hypoplastic RV | 1 (1.3) |
| ALCAPA repair | 1 (1.3) | | |

COA: aortic coarctation; VSD: ventricular septal defect; DORV: double-outlet right ventricle; AVSD: atrioventricular septal defect; ASO: arterial switch operation; TAPVC: total anomalous pulmonary venous connection; HAA: hypoplastic aortic arch; PA: pulmonary atresia; IAA:interrupted aortic arch; RV: right ventricle; ALCAPA: anomalous left coronary artery connected to the pulmonary artery; TGA: transposition of the great arteries.
